# Jonathan Taft

**Published:** 1903-11

**Authors:** 


### JONATHAN TAFT.

At midnight of the 15th of October, 1903, passed away from earth Dr. Jonathan Taft.

The life of such a man can scarcely be written by his contemporaries with justice to his memory. It has been too full of activities, in a professional sense, for more than a half-century, to be compassed in a few lines. Men die and their works live after them to enlighten and encourage those who have attempted to follow in their footsteps. Vain endeavor to be similarly gifted, for as no two are alike personally, so no man can be equally endowed, mentally and physically, with any other man.

Dr. Taft occupied a position in dentistry, if not original, at least unique in its character. He was not a devotee of what is termed scientific investigation; indeed, it may be doubted whether he was original, as that word is understood, yet he absorbed the work of other men so thoroughly that he became known throughout the country as an educator, with few, if any, equals. It was from this standard that his work must be judged. His life was so full of activity that the writer finds difficulty in realizing that Dr. Taft enjoyed a national reputation when he was a student, yet only ten years had elapsed since Dr. Taft had opened an office in Ripley, Ohio, in 1843.

Dr. Taft was not one to enter into warm disputations. His life was too serene, and his religious nature avoided the tempestuous conflicts that frequently disturbed the annual dental gatherings. He doubtless realized that storms and tempests are transitory and would end with a serener sky, and for this he could patiently wait. He was, therefore, never a partisan.

During the entire life of the American Dental Association he was a recognized authority. As far as the writer is aware, he never missed the annual gathering of this body, no matter how far the meeting might be from his home. When that body closed its work at Old Point Comfort, Dr. Taft, with much feeling, bid that body a long farewell. It had been his professional home for many years. One after another of his associates had passed to the life beyond, and while this body was to be reincarnated, as it were, into a new organization, he, with others, felt that he was too advanced in years to build up new associations. Notwithstanding this, he entered energetically into the work of the new organization, the National Dental Association, and has attended every meeting, including the last at Asheville, N. C.

He was one of the original organizers of the National Association of Dental Faculties, and has participated in its work up to the present year.

He took an early and active interest in dental education, and organized the Dental Department of Michigan University, and remained its Dean until the close of last session. It is not the time or place to traverse the action of this University in displacing him from the position he then held. Universities, like other large bodies, are not always grateful.

Dr. Taft, as a writer, did much to mould dental thought, but his work in this direction seemed rather the expression of other men's minds than that of his own. He failed to keep his work on "Operative Dentistry," first published in 1859, up to the standard of the passing years, and it was replaced by the text-books of other writers, but when first issued, and for a long time thereafter, served as a guide to the inexperienced student.

His nature was lovable and earnest. His whole life was devoted to his profession and his religious duties, and this he has left to those who still work in the ranks as a precious heritage. If his true professional spirit could enter into the life of dentistry to-day, how much better it would be for all who claim to be members of it as a profession. He belonged to a class of whom, unfortunately, we have too few, and the few are not appreciated.

While Dr. Taft had reached the limit of age ordinarily given to man, and while we cannot mourn his departure as we would the young, yet the world, and especially the dental world, has reason to regret that such as he cannot remain a blessing and an example to those most in need of such a gracious life. He was a warm friend, and as such the writer will miss him as the days go on, but with unfailing trust in the future that as men die men will live, and in the future the world will come to realize that death and life embody the idea of eternal progress.

The death of Dr. Taft was announced as this number was being prepared for the press, and therefore detailed notice of his life and work must be left for the December number.

